# Systematic review: cystic fibrosis in the SARS-CoV-2/COVID-19 pandemic

**DOI:** 10.1186/s12890-021-01528-0

**Published:** 2021-05-20

**Authors:** Hannah R. Mathew, May Y. Choi, Michael D. Parkins, Marvin J. Fritzler

**Affiliations:** 1 Department of Biological Sciences, University of Calgary, Calgary, AB Canada; 2 Cumming School of Medicine, University of Calgary, 3330 Hospital Dr. NW, Calgary, AB T2N 4N1 Canada

## Abstract

**Background:**

Severe acute respiratory syndrome coronavirus 2 (SARS-CoV-2) infection and the development of life-threatening COVID-19 are believed to disproportionately affect certain at-risk populations. However, it is not clear whether individuals with cystic fibrosis (CF) are at a higher risk of COVID-19 or its adverse consequences. Recurrent respiratory viral infections are often associated with perturbation and pulmonary exacerbations of CF as evidenced by the significant morbidity observed in CF individuals during the 2009 H1N1 pandemic. The primary goal of this review was to systematically survey published accounts of COVID-19 in CF and determine if individuals with CF are disproportionally affected by SARS-CoV-2 and development of COVID-19.

**Methods:**

We conducted a systematic literature search using EMBASE and Medline between April 28 and December 10, 2020. Six evaluable studies reporting on a total of 339 individuals with CF who developed COVID-19 were included in this study.

**Results:**

We found that although individuals with CF generally experience acute exacerbations of lung disease from infectious agents, COVID-19 incidence estimates in CF appear to be lower than in the general population. However, there are reports of subsets of CF, such as those who had organ transplants, that may experience a more severe COVID-19 course. Potential protective mechanisms in the CF population include pre-pandemic social isolation practices, infection prevention and control knowledge, altered expression of angiotensin-converting enzyme, and the use of certain medications.

**Conclusions:**

Although individuals with CF are at risk of acute exacerbations often precipitated by respiratory tract viral infections, published evidence to date indicated that individuals with CF do not experience higher risks of contracting SARS-CoV-2 infection. However, there is evidence that some subsets within the CF population, including those post-transplantation, may experience a more severe clinical course. As SARS-CoV-2 variants are identified and the pandemic goes through additional waves of disease outbreaks, ongoing monitoring of the risk of COVID-19 in individuals with CF is required.

## Introduction

Cystic fibrosis (CF) is a classic Mendelian autosomal recessive disorder and is the most common fatal genetic disease in North America [1]. The predominant incidence estimate of the disorder is 1/2500 live births in Caucasians, with a mean prevalence of 0.797/10,000 in the United States of America [2, 3]. The estimated median age of survival, which denotes the estimated age that 50% of infants born in a given year will live beyond, reaches 50 years and beyond in some countries [4]. CF has been traced to the inheritance of two abnormal copies of the CF transmembrane conductance regulator (CFTR) gene [1]. Abnormal CFTR are associated with aberrant salt and water transport across epithelial surfaces. Within the lungs, this manifests with mucus accumulation and the inability to clear inhaled organisms, giving rise to chronic infection and inflammation leading to airway remodelling and disease [1]. CFTR is also involved in the movement of bicarbonate; defects in its function lowers the pH of the airway surface liquid [1, 5]. Studies have shown this pH alteration in animal models impairs innate immunity by inhibiting the function of antimicrobial peptides [1, 6, 7]. Data also suggests that CFTR gene variants cause epithelial cells to inherently respond in an increasingly pro-inflammatory manner [1].

Severe acute respiratory syndrome coronavirus 2 (SARS-CoV-2) infection and the development of life-threatening COVID-19 are believed to disproportionately affect certain at-risk populations such as those over the age of 50 as well as those with diabetes, hypertension, cardiovascular diseases, and chronic respiratory disease [8–11]. However, it is not clear whether individuals with CF are at a higher risk of COVID-19 or its adverse consequences. This topic has been of great concern due to the importance of recurrent respiratory viral infections in disease perturbation and pulmonary exacerbations [12–14]. Additionally, significant morbidity was observed in individuals with CF during the 2009 H1N1 pandemic [15, 16]. The primary goal of this study was to survey published accounts of COVID-19 in CF and determine if CF constitutes an ‘at risk’ population with respect to morbidity or mortality from COVID-19.

## Methods

**Fig. 1:**
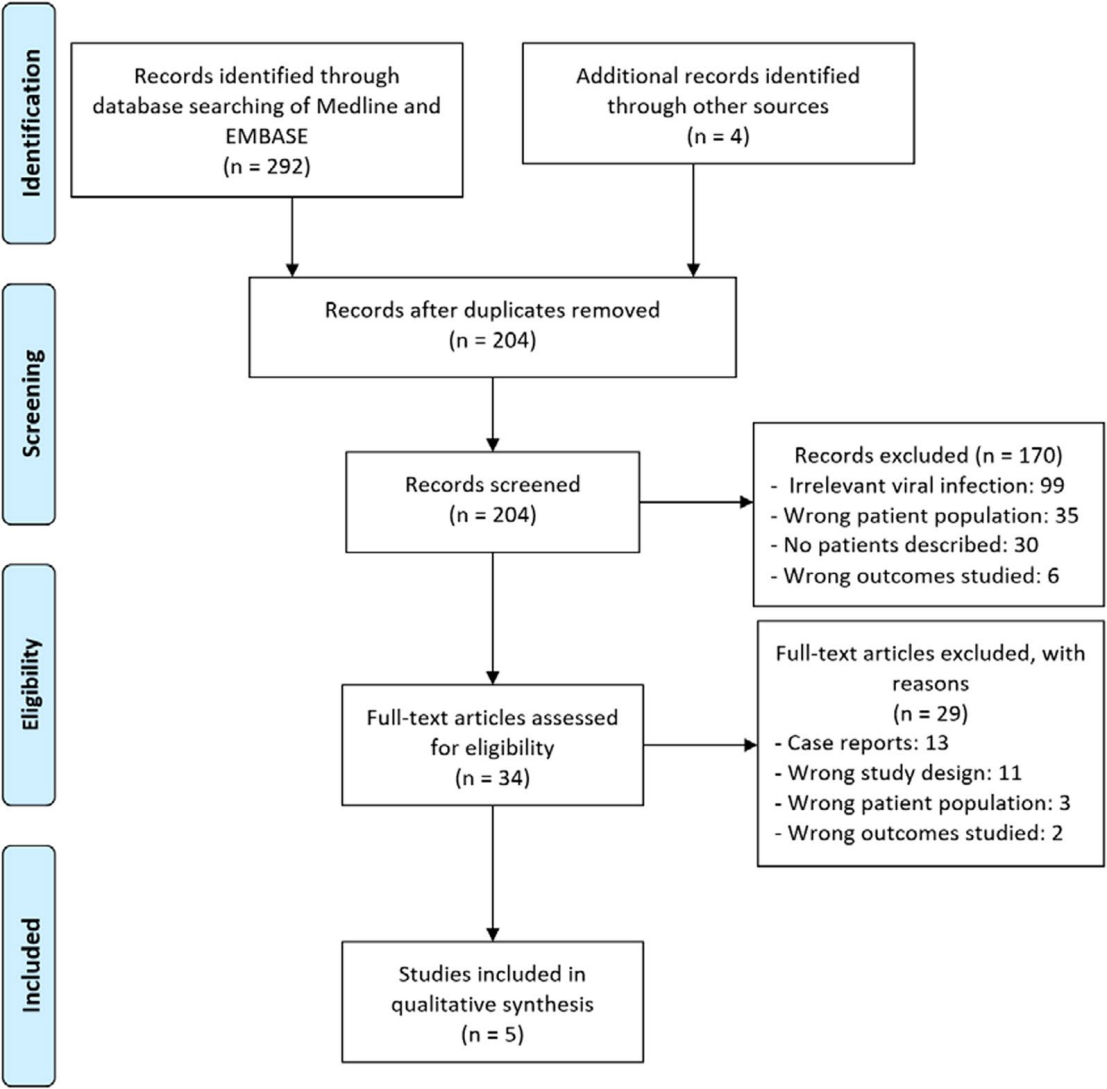
PRISMA flow diagram of literature identification, screening, assessment, and inclusion (http://prisma-statement.org/prismastatement/flowdiagram)

A literature search was conducted using EMBASE and MEDLINE between April 28 and December 10, 2020 (Fig. 1). The objective of the search was to identify studies that described COVID-19 disease outcomes in the population that contracted the virus, with exposure constituting a history of CF. The primary exclusion criteria consisted of singular case reports, absences of patient descriptions, and study of non-health-related outcomes. Both primary and secondary literature were included. Key search terms included “cystic fibrosis,” “coronavirus,” “SARS,” “SARS-CoV-2,” and “COVID-19” (see Table 1 for MEDLINE search strategy; see Table 5 for EMBASE search strategy). Each of the included studies was summarized (see Table 2) and evaluated using the Newcastle–Ottawa Quality Assessment Scale to examine risk of bias (see Table 3). Furthermore, information collected and reported by the European Cystic Fibrosis Society Patient Registry (ECFSPR) until April 16, 2021 on SARS-CoV-2 infection in CF was also included as a supplement to the extracted studies.

**Table 1:** MEDLINE electronic search strategy

Number	Search term(s)
1	Cystic fibrosis/
2	(Cystic fibrosis or fibrocystic disease* or mucoviscidosis).tw,kf
3	1 or 2
4	Exp coronaviridae infections/
5	Exp coronavirus/
6	(Severe acute respiratory syndrome coronavirus 2 or severe acute respiratory syndrome coronavirus or severe acute respiratory syndrome or covid-19 or covid19 or covid or coronavir* or corona vir* or ncov or novel coronavirus or novel cov or SARS-COV-2 or SARSCOV-2 or SARS-COV2 or SARSCOV2).tw,kf
7	4 or 5 or 6
8	3 and 7

**Table 2:** Clinical studies of COVID-19 in CF

Citation	Countries reporting	Sample size (n)	Study design	Main findings
Cosgriff et al. [18]	Australia, Canada, France, Ireland, Netherlands, New Zealand, UK, USA	40	Multinational cohort observational	Incidence of COVID-19 in CF was 0.07%, compared to 0.15% in general population
				Clinical features of COVID-19 similar in CF and general population
				No deaths in cohort
McClenaghan et al. [19]	Argentina, Australia, Belgium, Brazil, Canada, Chile, France, Germany, Ireland, Italy, Netherlands, New Zealand, Russia, South Africa, Spain, Sweden, Switzerland, UK, and USA	181	Multinational cohort observational	Post-transplant individuals (18%) and FEV_1_ < 70% were hospitalized at significantly higher rates
				7 deaths in cohort (3 post-transplant)
Corvol et al. [20]	France	31	Prospective cohort	Incidence of COVID-19 in CF was 0.41%, 93% less than general population
				Post-transplant individuals (39%) received supplemental oxygen and were hospitalized at significantly higher rates
				No deaths in cohort
Mondejar-Lopez et al. [21]	Spain	8	Retrospective descriptive cohort	Incidence of COVID-19 in CF was 0.32%, compared to 0.49% in general Spanish population
				Pediatric subset was not hospitalized, but all adults were
				No deaths in cohort
Bain et al. [22]	Argentina, Brazil, Chile, France, Germany, Italy, Russia, South Africa, Spain, Sweden, Switzerland, UK, US	105	Multinational cohort observational	29% asymptomatic
				FEV_1_ of hospitalized children significantly lower than non-hospitalized
				No deaths directly attributed to COVID-19 in cohort
Moeller et al. [23]	N/A^a^	14	Survey	7 individuals (50%) were hospitalized, with 3 in the PICU
				No deaths in sample

*CF* cystic fibrosis, *FEV*_*1*_ forced expiratory volume in 1 s, *PICU* pediatric intensive care unit, *UK* United Kingdom, *USA* Unites States of America.

^a^The survey stratified patients by asthma, cystic fibrosis, bronchopulmonary dysplasia, and other respiratory conditions but did not specify the number of countries for each condition

**Table 3:** Newcastle–Ottawa Quality Assessment Scale for cohort studies

	Selection (/☆☆☆☆)	Comparability (/☆☆)	Outcome (/✩✩✩)
Cosgriff et al. [18]	☆☆☆	☆	☆☆
McClenaghan et al. [19]	☆☆☆	☆	☆☆☆
Corvol et al. [20]	☆☆☆☆	☆	☆☆☆
Mondejar-Lopez et al. [21]	☆☆☆☆	☆	☆☆☆
Bain et al. [22]	☆☆	☆	☆☆
Moeller et al. [23]	☆☆☆	☆	☆☆

## Results

Six studies were included in this review, reporting on a total of 339 individuals with CF who developed COVID-19. Information collected by the European Cystic Fibrosis Society Patient Registry (ECFSPR) on SARS-CoV-2 infection in 1236 individuals with CF from 30 countries was also included [17].

### CF and COVID-19 studies

In a multinational cohort study of 40 individuals with CF from eight participating countries, the incidence of SARS-CoV-2 infection was 0.07%, which is approximately one-half of the 0.15% rate reported in the general population [18]. The median age of the infected cohort was 33 years, which was higher than the median age of the general CF population, with only one patient (3%) under the age of 16; this is reflective of the lower reportage of pediatric cases of COVID-19 diagnosed in the non-CF population [24]. The CF individuals in this cohort comprised of a heterogeneous population with various severe comorbidities and included those who were pregnant (3%) and had lung transplants (28%). The transplant recipient subgroup was 6 years post-transplant on average, with a range from 1 to 15 years. Of the 40 reported cases, 31 (78%) were symptomatic, and 24 (60%) were febrile at presentation. Thirteen (33%) received supplemental oxygen, four (10%) were admitted to the Intensive Care Unit (ICU), and one (3%) received invasive ventilatory support. The patient who received invasive ventilatory support was post-transplantation at the time of infection; however, information was not available on the number of years post-transplant or pre-infection immunosuppressant regimen. The clinical features of COVID-19 (e.g., fever, dry cough, myalgia) in CF appeared to be comparable to the general population affected by the SARS-CoV-2 virus.

Similar results were reported in a subsequently published multinational cohort study of 181 individuals with CF from 19 countries, including 40 from the previous study [19]. Thirty-two (18%) in the cohort were post-transplantation, with 28 (88%) lung transplantations, 2 (6%) liver transplantations, one (3%) lung and liver transplantation, and one (3%) lung and kidney transplantation. Eleven (6%) of the 181 individuals were admitted to the ICU, seven (64%) of whom were post-transplantation. Those individuals whose status was post-transplantation had a significantly higher risk of hospitalization, as evidenced by 74% of the post-transplantation subgroup and 46% of the non-transplantation subgroup requiring hospital care (*p* = 0.009). There were seven deaths (4%) in the cohort, three (43%) of whom were post-transplantation. Furthermore, individuals with a forced expiratory volume in 1 s (FEV_1_) less than 70% experienced significantly higher rates of hospitalization than those above 70% (*p* = 0.001).

In a study of 31 individuals with CF who acquired COVID-19 in France, the calculated incidence was 0.41%, which was 93% less than the general population at the time of the study [20]. The median age of the COVID-19 cohort was higher than that of the general CF population, which was reflective of the age-related findings of the 40-patient multinational cohort study [18]. Twelve individuals (39%) were post-transplant, and all were using oral corticosteroids in their immunosuppressant medication regimen. Three (10%) were asymptomatic; however, among the remaining 28, the most prevalent features at presentation were fever, fatigue, and worsened cough. Nineteen individuals within the cohort (61%) were hospitalized, and of the twelve (39%) who were post-transplantation—eleven (92%) were hospitalized. Of the four individuals (13%) that were admitted to the ICU, three (75%) were post-transplantation. Moreover, individuals who were post-transplantation received supplemental oxygen at significantly higher rates, as among seven of those (23%) who received supplemental oxygen therapy, six (86%) were post-transplantation. Eighteen (58%) individuals were on long-term azithromycin, with five (16%) receiving additional doses.

A study from Spain at the peak of the first pandemic wave reported eight CF individuals who acquired SARS-CoV-2 confirmed by reverse transcriptase-quantitative polymerase chain reaction (RT-qPCR) [21]. The study found the incidence of COVID-19 was 0.32% in their population of individuals with CF, which was less than the 0.49% rate in the general Spanish population. Two individuals (25%) were under 18 years of age, while the remaining six were adults. Neither of the two individuals under 18 were hospitalized, while all adults in the cohort did. Four of the adults (66%) received oxygen supplementation, but none received mechanical ventilation. Like the previous studies, one individual (13%) who was post-transplantation received intensive care support. Five in the cohort (63%) received azithromycin as part of their medical management. Ultimately, all eight of the described CF individuals recovered from infection.

Regarding the pediatric CF population, in a multinational cohort study of 105 CF children who had SARS-CoV-2 infection, the median age was 10 years and 2 (2%) were post-transplantation, with both making full recoveries [22]. The median best FEV_1_ among the 87 children in the cohort above the age of five within the 12 months leading up to infection was 94%. Of the 89 children for whom symptomatology data was available, 26 (29%) were asymptomatic, and the most common clinical features included fever and altered cough. COVID-19 symptoms in the cohort were similar to those reported for non-CF pediatric populations with SARS-CoV-2 infection. Of the 82 children for whom information on level of care was available, 24 (29%) were hospitalized, and one child in the cohort was admitted to the ICU (1%). Of the hospitalized patients whose respiratory support data was available, 6/21 (29%) received supplemental oxygen, 2/20 (10%) received non-invasive ventilation, and 1/20 (5%) received invasive ventilation. The median FEV_1_ of the children who were hospitalized was significantly lower than those who were treated at a community care level (*p* = 0.002). There were no deaths in the cohort directly ascribed to COVID-19. Thirty-one children (30%) were on long-term azithromycin.

In the responses to a survey sent to physicians of the Pediatric Assembly of the European Respiratory Society (ERS), 14 children with CF who acquired COVID-19 were briefly described [23], but there was no documentation of individuals post-transplantation in this sample. Four individuals (29%) experienced pulmonary exacerbation, five (36%) exhibited infection of the upper airway, two (14%) developed pneumonia, and one (7%) was merely febrile at presentation. Seven individuals in the pediatric cohort (50%) were hospitalized, with three of the seven (43%) admitted to the pediatric ICU and the remaining four (57%) to a pediatric ward. One individual (7%) received invasive ventilation therapy, and two (14%) received supplemental oxygen. There were no reported deaths within the sample population. Of the 14, three (21%) of the children received azithromycin as part of their infection treatment.

According to the ECFSPR, 1236 cases of COVID-19 in individuals with CF were reported in 30 European countries [17]. Of these, 946 (77%) cases were documented with at least partial data. The most prevalent age category was 18–29 years, and 56% had a FEV_1_ > 70. The most common symptoms were increased cough, fever, and fatigue. Of the 582 individuals with documented severities, 550 (95%) were mild or asymptomatic, 23 were (4%) severe cases, and 9 (2%) were critical. With respect to treatment, 217 individuals (23%) were hospitalized; 30 of these (14%) were admitted to the ICU. At the time of reporting, 866 (92%) individuals were fully recovered from infection, 39 (4%) had ongoing infection, and 13 (1%) died.

## Discussion

### COVID-19 incidence in CF

From the cases reported in the medical literature thus far, individuals with CF appear to be contracting SARS-CoV-2 viral infection at lower rates as compared to the general population [25]. Here and below, we discuss the factors that may mitigate COVID-19 in CF (summarized in Table 4). Most authors suspected that the lower rates of SARS-CoV-2 infection observed in individuals with CF relative to the general population are reflective of increased awareness of infection prevention and control practices, such as frequent hand hygiene and mask-wearing [26–28]. In addition, others suspected that rapid implementation and continued maintenance of physical and social distancing in CF populations were due to a perceived risk of COVID-19 respiratory disease exacerbation and complications [27, 28].

**Table 4:** Factors that may mitigate COVID-19 in cystic fibrosis

Category	Mitigating factor
Preventative measures	Social distancing/self-isolation
	Masking
	Frequent hand-hygiene
Physiological	Lower median age
	ACE and ACE2 expression
	Localized respiratory tract reduction of IL-6
	Altered interaction between SARS-CoV 3CL^pro^ and CFTR
	Thick secretions in respiratory tract
	Existing microbiota
	Elevated autophagy induction
Therapeutic/Interventional	Neutrophil elastase inhibitors
	Dornase alfa
	Azithromycin

*ACE* Angiotensin-converting enzyme, *ACE2* Angiotensin-converting enzyme 2, *IL-6* Interleukin-6, *3CL*^*pro*^ 3-chymotrypsin-like cysteine proteinase, *CFTR* CF transmembrane conductance regulator

### COVID-19 outcomes in CF

Despite demonstration that hospitalization rates are higher in CF than in the general population, individuals with CF also appear to have better outcomes than initially anticipated as compared to other respiratory viral infections [19]. From a pathogenic perspective, increased concentrations of neutrophil elastase are linked to enhanced lung damage and a decline in pulmonary function in CF [29]. Accordingly, neutrophil elastase inhibitors are being successfully used in trials to treat CF [29]. Of note, a pro-inflammatory imbalance of excess neutrophil elastase is involved in the development of acute respiratory distress syndrome (ARDS) associated with COVID-19 [30]. Therefore, neutrophil elastase inhibitors have also been proposed as potential therapies that could be repurposed for treating ARDS and the associated lung damage [31]. Furthermore, nebulized dornase alfa, a common CF medication, is currently undergoing trials for COVID-19 treatment [32]. Its proposed protective effect relates to its clearance of neutrophil extracellular traps, which play a pathogenic role in SARS-CoV-2 infection [33]. Of interest, preliminary data suggests that dornase alfa is effective in limiting the in vitro infection of green monkey and bovine kidney cell lines by SARS-CoV-2 [33]. Lastly, azithromycin, another commonly prescribed antibiotic in CF, has been suggested to potentially impact COVID-19—both by modulating the immune response and because of weak anti-viral activity [34, 35]. In early studies, the administration and use of chronic nebulized dornase alfa was not mentioned, but azithromycin use was described in four of the studies [20–23], either in regard to pre-existing use or as a treatment for COVID-19. In summation, it is possible that a CF patient exposed to SARS-CoV-2 might be on medications that may mitigate clinically severe COVID-19 (see Table 4) [36].

Since elevated interleukin-6 (IL-6) levels are associated with severe disease course and mortality in COVID-19, IL-6 is suggested to be an important cytokine in SARS-CoV-2 infection pathogenesis and ‘cytokine storm’ [37, 38]. However, in CF, there is a localized constitutive reduction of IL-6 in the respiratory tract, which suggests that it could serve as a protective factor from severe SARS-CoV-2 infection-related cytokine storms [37]. In a cohort of 39 individuals with advanced CF and chronic *Pseudomonas aeruginosa* infection, on average, the collected sputa contained high interleukin-8 (IL-8) levels and extremely low IL-6 and interleukin-10 (IL-10) levels [37]. This reduction of IL-6 was localized to the sputum, whereas systemic production of IL-6 was unaffected.

Angiotensin-converting enzyme 2 (ACE2) is the host cell receptor required for binding and entry of SARS-CoV-2 via the receptor-binding domain of the spike (S) protein [39]. SARS-CoV-2 infection causes *ACE2* transcriptional downregulation, resulting in an excessive synthesis of angiotensin II by angiotensin-converting enzyme (ACE) [40], a potential factor for exacerbated lung damage [41]. One study on an *ACE* biallelic polymorphism in 180 individuals with CF, termed “I” (insertion) and “D” (deletion), reported that subjects with the *ACE* D/D polymorphism were associated with an earlier appearance of clinical symptoms and higher risks of lung deterioration than those with I/I or D/I polymorphisms [42]. In another study on human coronaviruses (HCoV) in CF children, researchers did not find a significant association of HCoV infections with pulmonary exacerbations; however, those infected by HCoV-NL63, which also uses ACE2 for cell entry, had a significantly higher rate of pulmonary exacerbation than those with other coronavirus infections [43]. Taken together, the evidence suggests that a varied COVID-19 disease course in CF may in part be due to the association between *ACE* polymorphisms, effects on ACE2 downregulation, and resulting CF features, such as pulmonary inflammation [44].

It is also worth noting the importance of the SARS-CoV-2 3-chymotrypsin-like cysteine proteinase (3CL^pro^), which is critical in controlling viral replication and life cycle [45]. The SARS-CoV-2 3CL^pro^ shares 99.02% genomic similarity with the severe acute respiratory syndrome coronavirus (SARS-CoV) 3CL^pro^, and every residue involved in catalysis, dimerization, and substrate binding is entirely conserved [46]. As such, the 3CL^pro^ cleavage sites of the viral polyproteins are also considerably conserved [46]. The high degree of identity between SARS-CoV 3CL^pro^ and SARS-CoV-2 3CL^pro^ suggests that the activities of one are reflective of the other. The SARS-CoV 3CL^pro^ potential cleavage sites include the CFTR protein, which may be cleaved if available to the proteinase [47]. This information suggests that the cleavage of the CFTR protein may be involved in the molecular pathology of SARS-CoV-2, although the consequent effect on disease outcomes in the CF population is unclear.

It has also been postulated that thick secretions as well as the existing microbiota in the respiratory tract of individuals with CF may be protective mechanisms against viral infection [21]. Although information is limited, autophagy induction also plays direct and indirect roles in anti-viral response and is seen to be elevated in those with CF, suggesting its potential as a protective mechanism [48, 49].

According to the studies included in this review, history of solid-organ transplantation is hypothesized to be a potential determinant of COVID-19 disease outcomes. This finding is supported by another study describing 32 lung transplant recipients (transplanted for multiple aetiologies) who tested positive for COVID-19 [50]. In this study, 16% developed mild disease, 44% developed moderate disease, and 41% developed severe disease. Eighty-four percent were hospitalized, and 34% of the 32 individuals died, suggesting that COVID-19 is associated with a significantly higher mortality rate in lung transplant recipients than the general population. In this small study, the authors did not find a significant association between underlying pathology that necessitated lung transplantation (including CF) and the risk of severe COVID-19. Although certain immunosuppressant therapies in transplant recipients represent potential risk factors for severe disease [51, 52], baseline immunosuppressant doses were not significantly correlated with the severity of disease in the study. Indeed, because of the severe immune dysregulation culminating in a severe inflammatory component, immunomodulation and suppression strategies may be important if we are to modify the natural history of the later stages of COVID-19 [53–55]. Despite uncertainty regarding the mechanisms that elicit severe disease in this subgroup, the authors state that C-reactive protein, D-dimer, and IL-6 markers were disproportionally elevated in severe disease compared to moderate disease and may aid in determining disease prognoses in post-lung transplant individuals.

### Risk of bias assessment

Due to the unprecedented nature of the pandemic, we are indebted to the authors of these studies for their foresight in collecting and reporting data on the effect of SARS-CoV-2 infection in the CF population. However, the pandemic presented challenges in establishing certain elements of cohort studies, such as forming a distinct non-exposed cohort. While non-exposed (non-CF) participants were not recruited, an effort was made by some studies to compare results in the CF population to local statistics on the general population [20, 21]. Most studies were unable to control for factors beyond a method of defining a SARS-CoV-2 positive case, as the urgency for data on perceived at-risk populations, such as individuals with CF, prompted swift reporting [18–23]. The need for data early in the pandemic is also evidenced by the fact that, at the time of publication of the first multinational cohort study of 40 individuals, 30% of the cases were unresolved [18]; similarly, in the multinational cohort study of 105 CF children, two cases (2%) were unresolved at the time of publication [22]. The smallest study, which was a survey describing 14 children with CF who contracted COVID-19, had a low response rate; however, the results of the study were in accord with statistics reported by the larger studies included here [23].

### Limitations and future studies

Within the countries included in the analyzed studies, the reported cases of COVID-19 are not comprehensive and represent preliminary data. The data may be biased by the first wave of the pandemic, allowing for the possibility of the emergence of new information and trends over subsequent months. Furthermore, there is a lack of information regarding the long-term effects of COVID-19 disease in CF as well as the general population. Lastly, due to small sample sizes, univariate analyses were common among the analyzed studies and should be interpreted accordingly, especially considering univariate analysis could not be completed in the post-transplantation subgroup in the largest multinational cohort study published to date [19].

Further studies would benefit from analyzing the use of nebulized dornase alfa in those who developed COVID-19 and clarifying the uncertain effects of azithromycin in COVID-19 treatment. As well, the effect of ACE D/I biallelic polymorphism on COVID-19 severity in CF should be considered in future studies.

## Conclusions

Although individuals with CF are at risk of acute exacerbations of chronic lung disease, often precipitated by respiratory tract viral infections, published evidence to date indicates that incidence rates of SARS-CoV-2 infection may be lower in CF than the general population. Furthermore, data to date suggests that COVID-19 disease outcomes in individuals with CF may not be as severe as those brought on by H1N1 infection. However, there is evidence that some subsets within the CF population, including those post-transplantation, may experience a more severe clinical course. The limited cases of SARS-CoV-2 infection among the CF population is likely due to several factors that include effective physical and social distancing and the effective use of learned principles and practices of infection control stressed as parts of routine CF care. Other factors that are likely at play include altered expression of ACE, ACE2, and CFTR; and the use of medications with anti-viral effects.

## Data Availability

The datasets used and/or analysed during the current study are available from the corresponding author on reasonable request.

## Appendix

See Tables 5 and 6.

**Table 5:** EMBASE electronic search strategy

Number	Search term(s)
1	Exp cystic fibrosis/
2	(Cystic fibrosis or fibrocystic disease* or mucoviscidosis).tw,kw
3	1 or 2
4	Exp coronavirinae/ or exp coronavirus infection/
5	(Severe acute respiratory syndrome coronavirus 2 or severe acute respiratory syndrome coronavirus or severe acute respiratory syndrome or covid-19 or covid19 or covid or coronavir* or corona vir* or ncov or novel coronavirus or novel cov or SARS-COV-2 or SARSCOV-2 or SARS-COV2 or SARSCOV2).tw,kw
6	4 or 5
7	3 and 6

**Table 6:** Expanded Newcastle–Ottawa Quality Assessment Scale for Cohort Studies

	Selection				Comparability		Outcomes		
	Representativeness of exposed cohort	Selection of non-exposed cohort	Ascertainment of exposure	Demonstration that outcome of interest was not present at start of study	Study controls for most important factor	Study controls for other factor	Assessment of outcome	Follow-up length	Adequacy of follow-up
Cosgriff et al. [17]	☆	–	☆	☆	☆	–	☆	–	☆
McClenaghan et al. [18]	☆	–	☆	☆	☆	–	☆	☆	☆
Corvol et al. [19]	☆	☆	☆	☆	☆	–	☆	☆	☆
Mondejar-Lopez et al. [20]	☆	☆	☆	☆	☆	–	☆	☆	☆
Moeller et al. [21]	–^2^	☆	☆	☆	☆	–	☆	☆	–

## Abbreviations

ACE: Angiotensin-converting enzyme
ACE2: Angiotensin-converting enzyme 2
CF: Cystic fibrosis
IL-6: Interleukin-6
3CL^pro^: 3-Chymotrypsin-like cysteine proteinase
CFTR: CF transmembrane conductance regulator gene
FEV_1_: Forced expiratory volume in 1 s
ICU: Intensive care unit
IL: Interleukin cytokine
PICU: Pediatric intensive care unit
RT-qPCR: Reverse transcriptase-quantitative polymerase chain reaction
UK: United Kingdom
USA: Unites States of America
